# The Role of GDF15 in Regulating the Canonical Pathways of the Tumor Microenvironment in Wild-Type p53 Ovarian Tumor and Its Response to Chemotherapy

**DOI:** 10.3390/cancers12103043

**Published:** 2020-10-19

**Authors:** Daisy I. Izaguirre, Chun-Wai Ng, Suet-Yan Kwan, Eucharist H. Kun, Yvonne T. M. Tsang, David M. Gershenson, Kwong-Kwok Wong

**Affiliations:** 1 Department of Gynecologic Oncology and Reproductive Medicine, The University of Texas MD Anderson Cancer Center, Houston, TX 77030, USA; dizaguir@mdanderson.org (D.I.I.); CONg@mdanderson.org (C.-W.N.); suetyan.kwan@umassmed.edu (S.-Y.K.); Eucharistkun@utexas.edu (E.H.K.); ytsangle@mdanderson.org (Y.T.M.T.); DGershen@mdanderson.org (D.M.G.); 2 Cancer Biology Program, MD Anderson Cancer Center UTHealth Graduate School of Biomedical Sciences, Houston, TX 77030, USA

**Keywords:** ovarian cancer, *GDF15*, cisplatin, *p53*, tumor microenvironment, chemoresistance

## Abstract

**Simple Summary:**

Patients with wild-type *p53* ovarian cancer appear to have a poorer survival rate than those with mutant *p53* due to resistance to chemotherapy. The mechanism underlying this observation is not clearly understood. The aim of this study was to identify potential biomarkers regulated by p53 that conferred resistance using in vitro and in vivo studies. Growth differentiation factor 15 (GDF15) expression was demonstrated to be controlled by p53 in both ovarian cancer cell lines and orthotopic mouse models. The histological and RNAseq studies of the GDF15-knocked down, A2780 cell line-induced tumor revealed that the ratio and canonical pathways of stromal/tumor were modified by secretory GDF15.

**Abstract:**

Background: The standard treatment of ovarian cancer is surgery followed by a chemotherapeutic combination consisting of a platinum agent, such as cisplatin and a taxane-like paclitaxel. We previously observed that patients with ovarian cancer wild-type for *p53* had a poorer survival rate than did those with *p53* mutations. Thus, a better understanding of the molecular changes of epithelial ovarian cancer cells with wild-type *p53* in response to treatment with cisplatin could reveal novel mechanisms of chemoresistance. Methods: Gene expression profiling was performed on an ovarian cancer cell line A2780 with wild-type p53 treated with cisplatin. A gene encoding a secretory protein growth differentiation factor 15 (GDF15) was identified to be highly induced by cisplatin treatment in vitro. This was further validated in a panel of wild-type and mutant p53 ovarian cancer cell lines, as well as in mouse orthotopic models. The mouse tumor tissues were further analyzed by histology and RNA-seq. Results: GDF15 was identified as one of the highly induced genes by cisplatin or carboplatin in ovarian cancer cell lines with wild-type *p53*. The wild-type p53-induced expression of GDF15 and GDF15-confered chemotherapy resistance was further demonstrated in vitro and in vivo. This study also discovered that GDF15-knockdown (GDF15-KD) tumors had less stromal component and had different repertoires of activated and inhibited canonical pathways in the stromal cell and cancer cell components from that of the control tumors after cisplatin treatment. Conclusions: GDF15 expression from the wild-type *p53* cancer cells can modulate the canonical pathways in the tumor microenvironment in response to cisplatin, which is a possible mechanism of chemoresistance.

## 1. Introduction

Ovarian cancer is the fifth most common cause of death in women [1]. The standard treatment of ovarian cancer is surgery followed by a chemotherapeutic combination consisting of a platinum agent, such as cisplatin or carboplatin, and a taxane-like paclitaxel. Whereas the majority of high-grade serous ovarian cancers respond to treatment [2], several epithelial ovarian cancer subtypes with wild-type *p53* gene appear to be resistant to standard chemotherapy. For example, low-grade serous ovarian cancer is relatively resistant to various chemotherapy regimens [3]. Similarly, mucinous and clear cell ovarian carcinomas are also not very responsive to the standard taxane/paclitaxel combination [4,5]. We previously observed that patients with ovarian cancer wild-type for *p53* had a poorer survival rate than did those with *p53* mutations [6]. Thus, a better understanding of the molecular changes of epithelial ovarian cancer cells with wild-type *p53* in response to treatment with cisplatin could hopefully reveal novel mechanism of chemoresistance.

In the ovarian cancer cell line A2780, which is wild-type for *p53*, we found that growth differentiation factor 15 (GDF15) expression was highly induced by cisplatin exposure. Expression of this gene is known to be induced by factors such as cellular stress, inflammation, and exposure to nonsteroidal anti-inflammatory drugs [7,8,9,10,11,12,13]. GDF15 is also known as nonsteroidal anti-inflammatory drug-activated gene, macrophage inhibitory cytokine-1, placental bone morphogenic protein, placental transformation growth factor-β, and prostate-derived growth factor [14,15,16,17]. GDF15 belongs to the transforming growth factor-β superfamily, and its receptor (GFRAL) was recently identified [18,19]. The GDF15/GFRAL pathway is known to affect energy homeostasis, which could have implications for cancer initiation and progression [20].

In previous studies, *GDF15* expression was elevated in many cancers such as breast, cervical, colorectal, ovarian, pancreatic, and prostate cancers as well as glioblastomas, melanomas, and oral squamous cell carcinomas [21,22,23,24,25,26,27,28]. As seen in these cancers, high expression of *GDF15* is associated with poorer survival than in patients with low expression [23,24]. However, depending on tumor type, *GDF15* has been shown to be either protumorigenic or antitumorigenic. For example, its overexpression in glioblastoma and human colon cancer cells inhibited tumor formation in nude mouse models [8,10]. In contrast, another study showed that overexpression of *GDF15* led to an increase in the dissemination of prostate cancer cells [29].

In the present study, we performed gene expression analysis of the ovarian cancer cell line A2780 with wild-type *p53* treated with 5 or 10 μM cisplatin at different time points. In parallel, gene expression study was also done with the ovarian cancer cell line OVCA420 with mutant *p53*. GDF15 was among the most differentially expressed genes in the cells with wild-type *p53* but not in mutant *p53* cells. Using a panel of ovarian cancer cell lines and orthotopic mouse models of ovarian cancer, we would like to demonstrate that the level of *GDF15* expression induced by chemotherapeutic agents is p53-dependent and that up-regulation of secretory GDF15 protein by treatment with cisplatin can be detected in the mouse sera. Our mouse models allowed us to detect GDF15 secretion into the blood and the impact of GDF15 level on the tumor and stroma interaction in vivo. More importantly, we proved that GDF15 expression impacts ovarian tumorigenesis by modulating the canonical pathways in both the tumor cell and stroma cell components of the tumor.

## 2. Results

### 2.1. Differential Expression of Genes in the Cisplatin-Sensitive, Wild-Type p53 Ovarian Cancer Cell Line A2780—Following Cisplatin-Based Treatment

**Figure 1:**
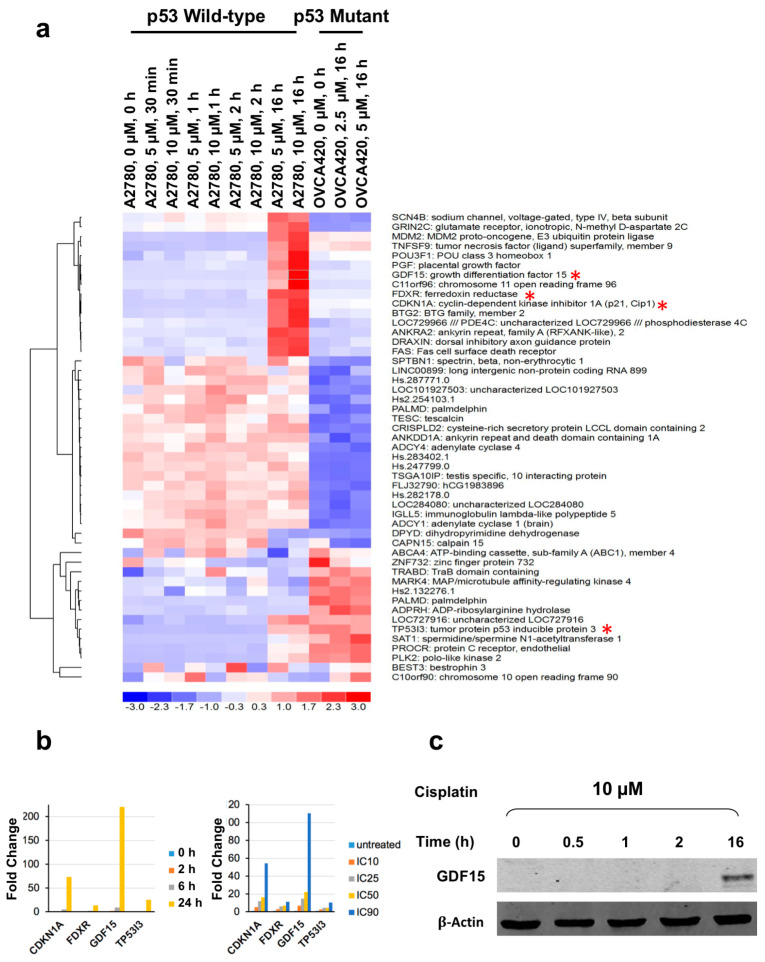
Differentially expressed genes identified by microarray analysis in wild-type *p53* A2780 ovarian cancer cell line treated with cisplatin: (**a**) Heat map of genes with differential expression in the A2780 ovarian cancer cell line induced by treatment with cisplatin. The expression of these genes in the *p53* mutant OVCA420 cell line was also shown. The color bar at the bottom indicates the fold change in reference to the expression value at zero hour on log2 scale; * indicated the genes validated in (**b**); (**b**) Validation of the up-regulation of the four genes by semi-quantitative RT-PCR analysis performed using total RNA extracted from A2780 cells treated with cisplatin at increasing doses (2.5, 4.5, 5.75, and 25 μM) for 16 h or a single dose at IC90 for different times (in triplicate); (**c**) Western blot showing expression of GDF15 in A2780 cells that were treated by cisplatin as indicated.

To identify genes potentially involved in cisplatin response of ovarian tumors with wild-type p53, we conducted gene expression profiling of the A2780 cell line (wild-type p53) and OVCA420 (mutant p53; c.818G > A; p.R273H). We have deposited the microarray data into GeoArchive (GEO accession #GSE122287). Figure 1a shows the most differentially expressed genes 16 h after cisplatin exposure. We chose four genes (*CDKN1A*, *FDXR*, *GDF15*, and *TP53I3*) with the highest expression levels and fold induction for further validation at the RNA and protein level (Table S1). These genes did not change in the OVCA420 *p53* mutant cell line after treatment with cisplatin (Figure 1). Previous study showed that *BTG2* expression in prostate cancer cells was induced by treatment with cisplatin [30], so we did not choose it for further investigation. The RNA expression for all four genes was induced in a time- and dose-dependent manner (Figure 1b). The robust induction of GDF15 protein was detected after 16 h of cisplatin treatment (Figure 1c).

### 2.2. Induction of GDF15 Expression in a Panel of Ovarian Cancer Cell Lines by Exposure to Chemotherapeutic Agents

**Figure 2:**
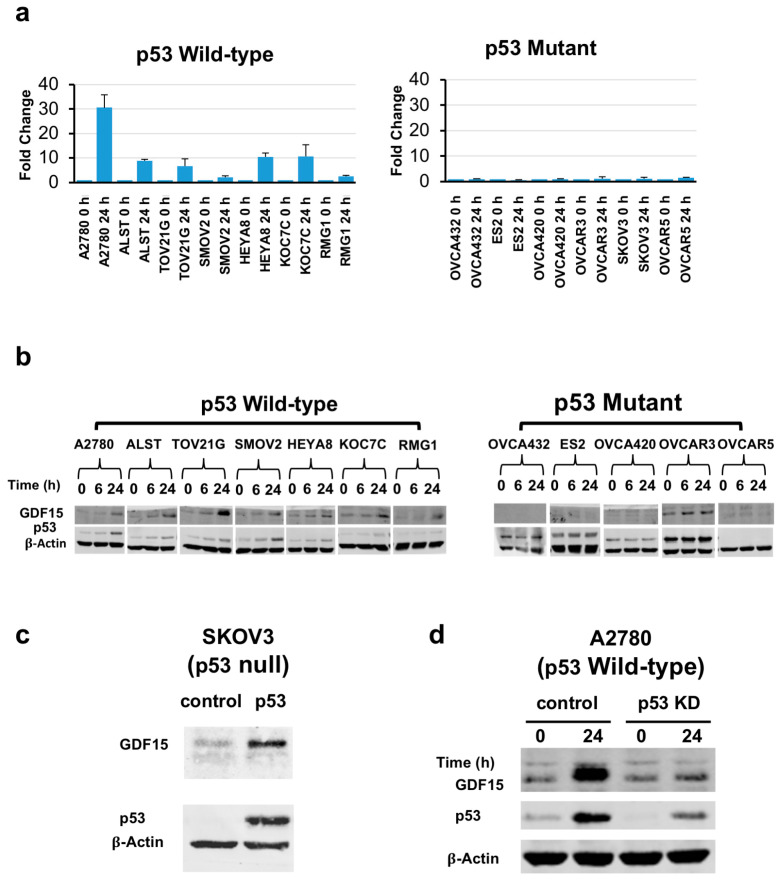
Induction of GDF15 expression in ovarian cancer cell lines after treatment with cisplatin depends on the presence and induction of wild-type p53. Cell lines with wild-type *p53* or mutant *p53* were treated with cisplatin at IC90 dose for 24 h (2.5 μM: A2780, OVCA432; 5 μM: ALST, OVCAR3, OVCA420, TOV21G, SMOV2; 10 μM: ES2, HEYA8, KOC7C, OVCAR5, RMG1, SKOV3): (**a**) Semiquantitative RT-PCR analysis of *GDF15* expression using total RNA extracted from a panel of ovarian cancer cell lines with wild-type *p53* or mutant *p53* confirmed by PCR-Sanger sequencing (in triplicate); (**b**) Western blots of GDF15 and p53 protein expression in ovarian cancer cell lines after treatment with cisplatin; (**c**) GDF15 was induced when SKOV3 ovarian cancer cells (p53-null) were transduced with an adenovirus carrying the wild-type *p53* gene; (**d**) Knockdown of *p53* (p53 KD) expression in A2780 ovarian cancer cells by *p53* small interfering RNA suppressed the induction of GDF15 protein expression by treatment with cisplatin.

Among the four validated genes described above, we chose *GDF15* for further analysis as it had the highest induced expression following treatment with cisplatin. To determine whether our observation was specific to A2780 cells, we further treated 13 ovarian cancer cell lines (7 wild-type for p53 and 6 with missense or nonsense p53 mutations) with 2.5 μM cisplatin (Figure 2). RNA and protein analysis of these cells revealed that GDF15 up-regulation occurred only in the cell lines with wild-type *p53* (Figure 2a,b), suggesting that the up-regulation of GDF15 following cisplatin treatment could be a p53-dependent event. Original uncropped western blots images were provided in Figure S4.

**Figure 3:**
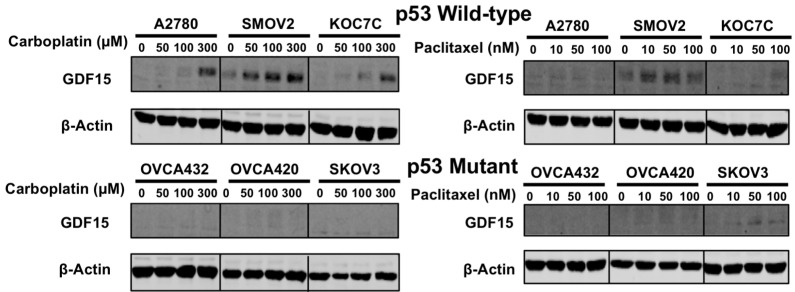
GDF15 expression in ovarian cancer cell lines after treatment with carboplatin or paclitaxel. Cell lines with wild-type *p53* (A2780, SMOV2, KOC7C) or mutant *p53* (OVCA432, OVCA420, SKOV3) were treated with varying doses of carboplatin or paclitaxel for 24 h.

Ovarian cancer treatment consists of debulking surgery followed by administration of a combination of chemotherapeutic agents, typically a taxane-like paclitaxel and a DNA-damaging agent such as cisplatin or carboplatin. Therefore, we further treated six ovarian cancer cell lines (three wild-type for *p53* and three with *p53* mutations) with varying doses of carboplatin or paclitaxel. Similar to the cell lines treated with cisplatin, the cell lines wild-type for *p53* had up-regulation of *GDF15* following treatment with carboplatin, whereas *GDF15* expression was not affected in the p53-mutant cell lines. On the other hand, paclitaxel did not induce GDF15 expression in either p53 wild-type or p53 mutant ovarian cancer cell lines (Figure 3).

### 2.3. Induction of GDF15 Expression Requires Wild-Type p53

To further determine whether GDF15 expression is dependent on *p53* following cisplatin-based treatment, we infected the p53-null cell line SKOV3 (confirmed by western blot and Sanger sequencing to have a single base deletion in exon 4 of p53 [31]) with an adenovirus containing the wild-type p53 gene as described previously [32]. Following p53 overexpression in the cell line, we observed higher expression of GDF15 than that in control cells (Figure 2c). Furthermore, knockdown of p53 expression by small interfering RNA in A2780 cells wild-type for *p53* suppressed the induction of GDF15 expression by cisplatin exposure in comparison to the control cells (Figure 2d).

### 2.4. GDF15 Is Secreted into the Blood In Vivo in Mice with Tumor Burdens after Treatment with Cisplatin

**Figure 4:**
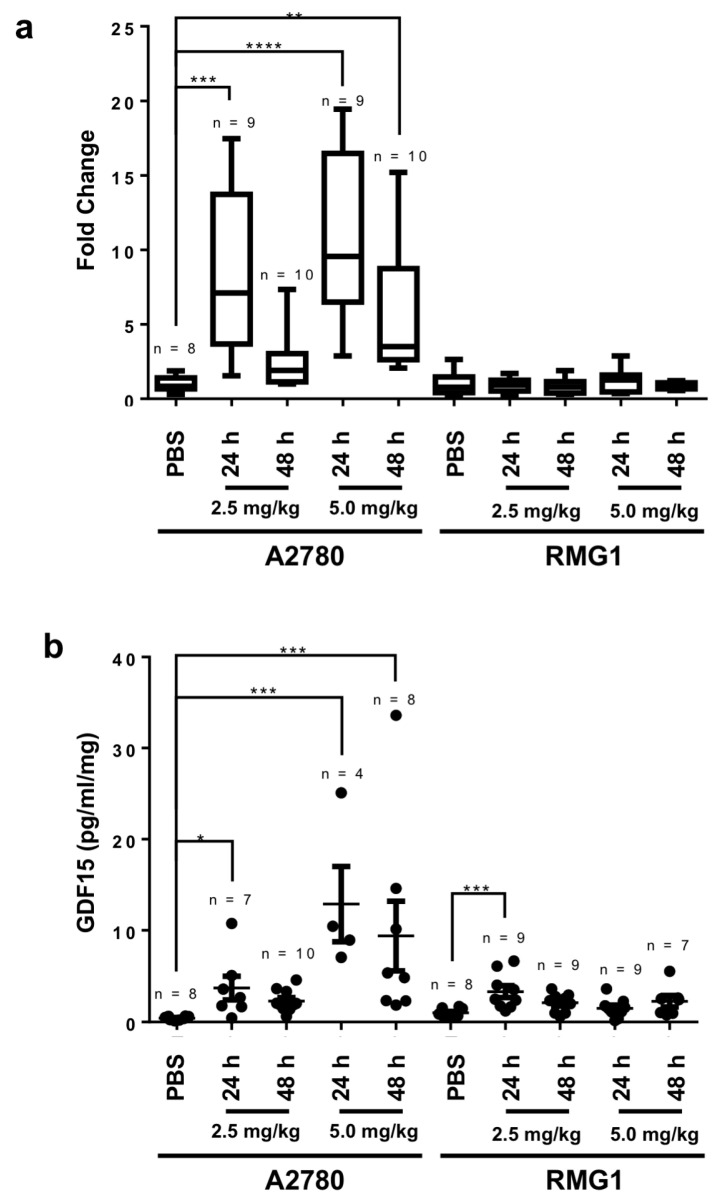
Induction of GDF15 expression in mouse ovarian tumors and sera by cisplatin treatment in vivo: (**a**) Semiquantitative RT-PCR analysis of *GDF15* expression performed using total RNA extracted from mouse A2780 (cisplatin-sensitive) and RMG1 (cisplatin-resistant) tumors after treatment with cisplatin (2.5 or 5 mg/kg) for 24 or 48 h. **p = 0.004; ***p = 0.0007; ****p < 0.0001; (**b**) Enzyme-linked immunosorbent assay of GDF15 sera levels in mice. *p = 0.03; ***p = 0.0002.

Because mature GDF15 is a secreted protein, we further examined its expression in the blood in vivo following cisplatin-based treatment. We chose two *p53* wild-type ovarian cancer cell lines for this mouse experiment: A2780 (cisplatin-sensitive, IC_50_ = 1.3 μM) and RMG1 (cisplatin-resistant, IC_50_ = 7.5 μM). After tumors were formed in these mice, they were treated with phosphate-buffered saline (PBS) or 2.5 or 5 mg/kg cisplatin. We then collected the tumors and blood from these mice 24 and 48 h after treatment. Figure 4 shows the results of a combination of two independent experiments using mice with different tumor burdens. The data demonstrated that mice given cisplatin had a significant up-regulation of *GDF15* at the RNA level in tumors (Figure 4a) and/or sera (Figure 4b). As a control to ensure the specificity of the enzyme-linked immunosorbent assay for the anti-human GDF15 antibody, we detected no GDF15 protein in sera from control mice without any ovarian tumor (data not shown). More importantly, up-regulation of GDF15 was much higher in the mice injected with the cisplatin-sensitive A2780 cell line than in the mice injected with the cisplatin-resistant RMG1 cell line (Figure 4).

### 2.5. GDF15-KD Increases Tumor Burden and Tumor Sensitivity to Cisplatin-Based Treatment In Vivo

To determine the role of GDF15 in the response of ovarian cancer to treatment with cisplatin, we generated stable GDF15-KD A2780 cell lines using two different shRNAs targeting GDF15. As a control, we also generated a stable control GDF15-NT A2780 cell line using non-target shRNA (NT). The efficiency of GDF15 knockdown was confirmed in vitro and in vivo by real-time polymerase chain reaction (RT-PCR), western blot and enzyme-linked immunosorbent assay (Figure S2). Then, we intraperitoneally injected nude mice with control GDF15-NT A2780 or GDF15-KD A2780 cells. Once tumors developed, the mice were given either 5 mg/kg cisplatin or PBS twice within a 5-day period. Forty-eight hours after the last treatment, we obtained the tumors and blood from the mice.

**Figure 5:**
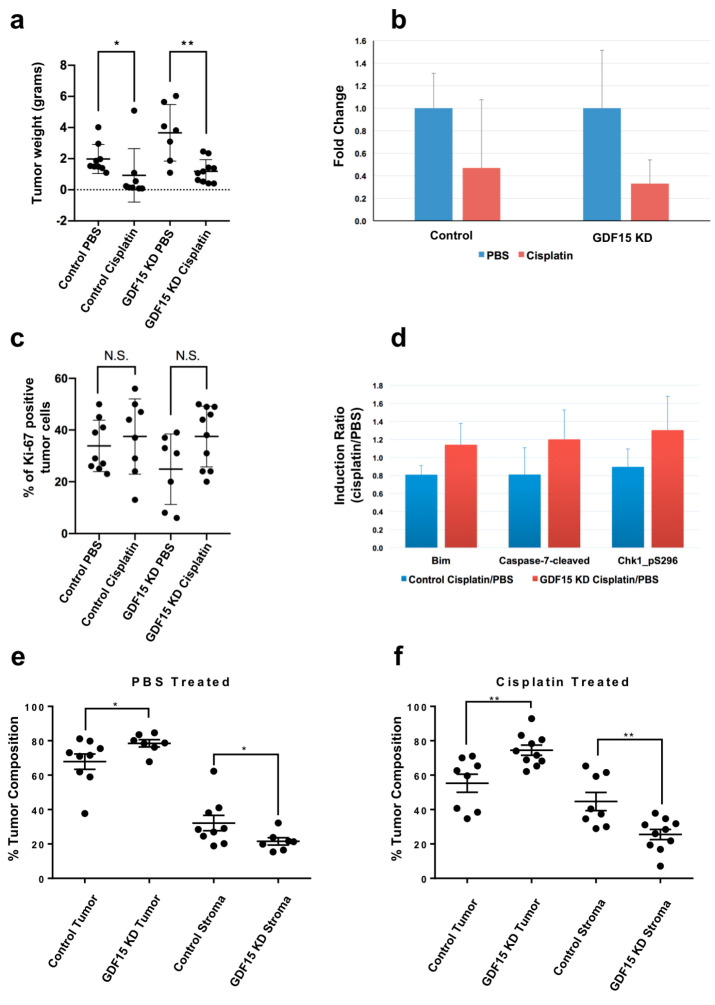
The impact of GDF15 KD on ovarian tumor growth, cisplatin response and tumor composition; (**a**) Tumor weights in mice after treatment with PBS or cisplatin. * *p* = 0.0079; ** *p* = 0.0046; (**b**) Fold decrease in tumor weights after treatment with cisplatin normalized to control tumors treated with PBS; (**c**) Percentage of Ki67-positive ovarian tumor cells in the tumor. N.S., not significant; (**d**) RPPA analysis results showing induction of higher expression of the apoptotic proteins Bim, cleaved caspase-7, and phosphorylated Chk1 after treatment with cisplatin in GDF15-KD A2780 tumors compared to normal A2780 tumors; (**e**) Percentage of tumor and stromal cells in mouse A2780 and GDF15-KD A2780 tumors after treatment with PBS; (**f**) Percentage of tumor and stromal cells in mouse A2780 and GDF15-KD A2780 tumors after treatment with cisplatin.

Tumor weight analysis revealed that the GDF15-KD tumors were substantially larger than the control tumors (Figure 5a). Also, cisplatin-treated mice in both the control and GDF15-KD groups had smaller tumors than did PBS-treated mice. To determine whether GDF15 expression makes the tumors more sensitive or resistant to treatment with cisplatin, we normalized the weights of the cisplatin-treated tumors to the weights of the PBS-treated tumors (Figure 5b). This revealed that the decrease (fold change) in the size of the GDF15-KD tumors (67%) after cisplatin-based treatment was significantly greater than that in the control (53%) (Figure 5b).

Because tumor growth is a balance between proliferation and apoptosis, we sought to determine whether cell proliferation differed in GDF15-KD mice compared to the control following cisplatin exposure. We performed immunohistochemical staining of ovarian tumor samples treated with cisplatin for Ki67, a marker of cell proliferation. As a result, the percentage of Ki67-positive tumor cells after cisplatin treatment did not differ significantly (Figure 5c) from untreated control. However, reverse-phase protein array (RPPA) analysis demonstrated that expression of proteins involved in apoptosis (Bim, cleaved caspase-7, and phosphorylated Chk1) was markedly higher in cisplatin-treated GDF15-KD A2780 tumors than the control A2780 tumors (Figure 5d).

### 2.6. GDF15 KD Affects Tumor Composition after Cisplatin-Based Treatment

As a secreted protein, GDF15 could have both autocrine and paracrine signaling. To determine whether GDF15 affects tumor stroma composition, we used microscopic images of Ki67 stains for cellular segmentation analysis of tumor and non-tumor cells within the tumors from both GDF15-KD and control mice (Figure S1). Based on cellular morphology and Ki-67 staining, the tumor tissue was segmented into tumor cell regions (Ki-67 positive cells and/or pleiomorphic nuclei) and stromal regions (vascular/fibrous region, other non-tumor cell type region and noncellular region). Quantification of the tumor and stromal compartments demonstrated that the percentage of the stromal cell component of the tumors was lower in GDF15-KD tumors than in control tumors in both the PBS- and cisplatin-treated groups (Figure 5e,f). However, after treatment with cisplatin, the stromal cell component appeared to be higher in the control GDF15-NT A2780 tumors than the GDF15-KD A2780 tumors (Figure 5f). Because the stromal cells in the A2780 tumors were derived from mice, we measured the relative abundance of mouse tissue in tumors formed by GDF15-NT A2780 and GDF15-KD A2780 cells indirectly using mouse IgG detection signals as a surrogate of stromal composition using RPPA data. In the RPPA analysis, we had 60 mouse anti-human primary antibodies; thus, the secondary anti-mouse IgG antibody would recognize any endogenous mouse IgG. We compared the relative signals detected using these 60 antibodies and found that GDF15-KD A2780 tumors had significantly decreased signals (Figure S3). This demonstrated that the endogenous mouse IgG levels were lower in tumors formed by GDF15-KD A2780 cells than in the control, and thus the stromal composition was lower in GDF15-KD A2780-induced tumors than the GDF15-NT-induced tumors.

To further investigate the impact of knocking down *GDF15* in the tumor formation by A2780 in nude mice, we performed RNAseq analysis of GDF15-NT A2780 and GDF15-KD A2780 tumors from mice treated with PBS or cisplatin. We performed a novel RNAseq analysis by mapping the RNAseq data to both the human reference genome and mouse reference genome. This allowed us to interrogate the gene expression changes in both A2780 ovarian cancer cells (human) and stromal cells (mouse) by GDF15 knockdown and cisplatin treatment. We found that there was a substantial increase in the number of sequencing reads that mapped to the mouse reference genome (Table S2) in GDF15-NT A2780 control tumors after cisplatin treatment (PBS treated, 17 ± 2% versus cisplatin treated, 37 ± 16%). On the other hand, only a small change in the number of sequencing reads that mapped to the mouse reference genome was observed in GDF15-KD A2780 after cisplatin treatment (PBS treated, 18 ± 0% versus Cisplatin treated, 19 ± 2%). This would suggest an increase in the stromal component contributed by the mouse in cisplatin treated GDF15-NT A2780 tumors. This result also agreed with the cellular segmentation analysis that cisplatin treated control GDF15-NT A2780 tumor had more stromal component (Figure 5e,f).

**Figure 6:**
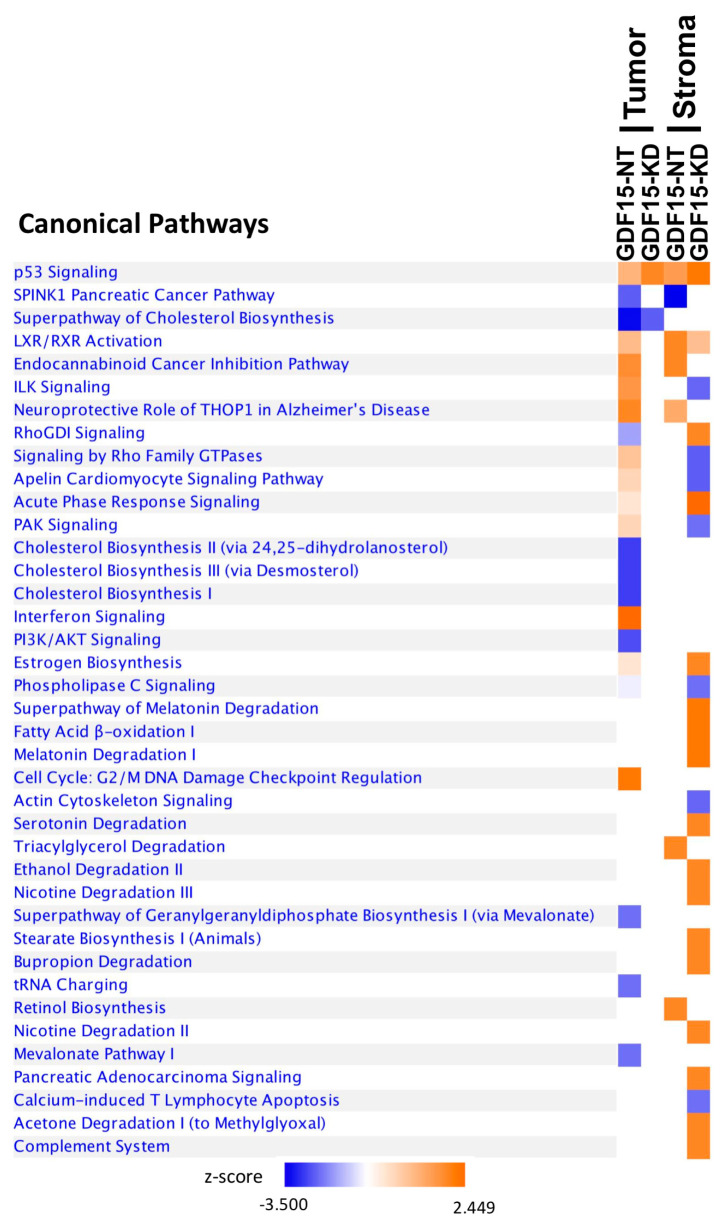
Activation or inhibition of canonical pathways in the tumor cell and stromal cell components of tumors formed by A2780 GDF15-NT control cells or A2780 GDF15-KD cells by cisplatin treatment. Z-score is a statistical measure of the match between expected relationship direction and observed gene expression changes after cisplatin treatment. z-score > 2 (activation) or z-score < 2 (inhibition) are considered significant.

Both human and mouse genes differentially expressed in the cisplatin and PBS-treated groups are provided in Tables S3–S6. Ingenuity Pathway Analysis (IPA) was used to identify the changes in canonical pathways that may be responsible for gene expression changes in GDF15-NT and GDF15-KD after cisplatin treatment. As expected, the canonical p53 pathways are highly activated in both GDF15-NT and GDF15-KD tumors after cisplatin treatment in both the tumor cell and stromal cell components (Figure 6). However, several activated canonical pathways were not activated in the tumor component of GDF15-KD in comparison to GDF15-NT (Figure 6) such as the interferon signaling pathway and cell cycle: G2/M DNA damaging checkpoint regulation pathway, which suggests GDF15 expression may be involved in those pathways. On the contrary, there are several canonical pathways being activated in the stromal cell component of GDF15-KD tumor after cisplatin treatment compared to the GDF15-NT control such as the RhoGDI signaling pathway and the acute phase response signaling pathway.

## 3. Discussion

In this study, we showed that GDF15 expression is induced in ovarian cancer cells by treatment with the chemotherapeutic agent cisplatin both in vitro and in vivo. Furthermore, our data demonstrated that induction of GDF15 expression in ovarian cancer cells by treatment with platinum agents is dependent on the presence of wild-type p53. This observation is more relevant to chemoresistant ovarian carcinomas with wild-type p53, such as low-grade serous ovarian carcinomas, clear cell ovarian carcinomas, and most of the endometrioid and mucinous ovarian carcinomas, than to high-grade serous ovarian carcinomas which tend to be mutant for p53.

We also showed that GDF15 can be detected in sera from ovarian tumor-bearing mice after cisplatin exposure. For this mouse experiment, we used two *p53* wild-type ovarian cancer cell lines: A2780 (cisplatin-sensitive) and RMG1 (cisplatin-resistant). The A2780 tumors had at least 15-fold higher induction of *GDF15* expression than did the RMG1 tumors when treated at 2.5 µM cisplatin. The discrepancy of *GDF15* induction in RMG1 cisplatin-resistant cell line by cisplatin in vitro and in vivo was likely the result of the doses of cisplatin being used. For in vitro study, when RMG1 was treated at IC90 dose (10 μM), an up-regulation of *GDF15* was detected. However, for in vivo study, only up to 5 mg/kg cisplatin dose was used because the known maximum tolerated dose of cisplatin for mice is 6 mg/kg. However, at such a dose, up-regulation of GDF15 was not detected in tumor formed by the cisplatin-resistant RMG1 cell line in mice. A previous ovarian cancer study showed that GDF15 expression was higher in post-chemotherapy than in pre-chemotherapy effusions [33]. Because low-grade serous ovarian carcinoma is relatively resistant to chemotherapy [34], developing GDF15 as a potential serum biomarker to select patients that are sensitive to chemotherapy warrants further investigation.

Using our orthotopic mouse model of ovarian cancer in which *GDF15* was knocked down enabled us to look at GDF15 from two angles: its role in ovarian tumorigenesis and its effect following treatment with cisplatin. As noted above, GDF15-KD A2780 cells formed larger tumors in nude mice than did GDF15-NT A2780 control cells, suggesting that basal level of GDF15 affects tumor growth. Ki67 staining is commonly used as a surrogate marker for tumor cell proliferation. However, we did not observe any significant increase in the percentage of Ki67-positive tumor cells in mice after GDF15 KD or a decrease in the percentage of Ki67 after treatment with cisplatin. A previous study suggested that Ki67 can be expressed in growth-arrested cells, especially those overexpressing wild-type p53 [35]. In another study, researchers compared Ki67 expression in breast tumor samples before and after chemotherapy. Only 40% of the tumors exhibited a significant decrease in Ki67 expression [36]. This may have been the case with our ovarian tumors formed by A2780 cells wild-type for p53, in which Ki67 expression did not correlate with tumor cell proliferation. After tumor bearing mice were treated with cisplatin treatment, the decrease in tumor weight was more drastic in the GDF15-KD A2780 tumors than the control GDF15-NT A2780 tumors, suggesting that GDF15 induction increases tumor resistance to cisplatin. Our RPPA analysis demonstrated that expression of phosphorylated Chk1 protein was more highly induced in GDF15-KD A2780 tumors than in control tumors. Since phosphorylation of Chk1 can induce checkpoint-mediated cell-cycle arrest [37], this might be one of the mechanisms why GDF15-KD A2780 tumors appeared to be more sensitive to cisplatin treatment than the control tumors.

In addition, we showed that GDF15 expression can impact the response of both the tumor and stromal cells to cisplatin. GDF15-KD tumors had less stromal component than did control tumors. Moreover, following cisplatin-based treatment, the control tumors with higher induced GDF15 had a higher stromal component, whereas the GDF15-KD tumors did not have a significant change in the tumor-stroma ratio, suggesting that GDF15 secreted by tumor cells can protect or recruit stromal cells against this treatment. This might have indirectly made the tumor cells more resistant to the treatment. The tumor microenvironment is known to affect tumor response to chemotherapy. Specifically, a recent study demonstrated the recruitment of myeloid cells to the tumor in a mouse mammary tumor model following treatment with doxorubicin [38] and GDF15 can enhance the tumor-initiating and self-renewal potential of multiple myeloma cells [39]. Stromal GDF15 has also been identified by others to be involved in the malignancy of ovarian cancer [40]. Although additional studies are needed to identify the stromal component affected by GDF15 expression as well as how GDF15 might recruit stromal cells, we are the first to show the effect of wild-type p53 in ovarian cancer cells on GDF15 expression and the impact of GDF15 on the stroma components. More significantly, using a novel strategy to interrogate the gene expression change in both tumor cell and stroma cell component of the mouse tumor, we were able to identify novel pathway changes in the stromal component after cisplatin treatment. As shown by the canonical pathway comparative analysis of GDF15-NT and GDF15-KD mouse tumor and stroma, pathways that could be responsible for the crosstalk were identified. For instance, in GDF15-NT tumor, interferon signaling, and G2/M DNA damage checkpoint regulation were highly up-regulated after cisplatin treatment. This suggests that GDF15 could be an important factor in tumor progression by enhancing interferon signaling and DNA repair of tumor cells. While in the GDF15-KD stroma, various degradation pathways were activated, including the degradation of melatonin, serotonin, ethanol, nicotine, acetone, and bupropion, which might play a role in chemosensitivity of this tumor. Since the activation of degradation pathways were only present in GDF15-KD stroma, this might indicate that these cells were undergoing a process of apoptosis, which could explain the decrease in the stromal composition in GDF15-KD tumor.

It has been previously shown that secretome of tumor cells induced by therapy treatment can promote resistance and tumor progression [41], and GDF15 could be one of the secreted proteins in the context of cisplatin resistance. The crosstalk between secreted GDF15 and the stromal environment may play an important role in developing drug resistance by remodeling the tumor microenvironment as have been observed in other environment-mediated drug resistance [42]. Since the receptor of GDF15 has been discovered [18], targeting the GDF15 receptor could be a potential therapeutic strategy to overcome chemoresistance.

## 4. Materials and Methods

### 4.1. Cell Lines

The following ovarian cancer cell lines were used in this study: A2780, ALST, ES2, HEYA8, KOC7C, OVCAR-3, OVCAR-5, OVCA420, OVCA432, RMG1, SKOV3, SMOV2, and TOV21G. For in vivo experiments, RMG1 and A2780 cells containing a luciferase vector (RMG1-luc and A2780-luc) were used. All cell lines were cultured in RPMI medium (Sigma-Aldrich, St. Louis, MO, USA) containing 10% fetal bovine serum (Invitrogen, Carlsbad, CA, USA), 100 U/mL penicillin, and 100 μg/mL streptomycin (Sigma-Aldrich, St. Louis, MO, USA) in a humidified incubator at 37 °C in a 5% CO_2_ atmosphere. A2780 and ES2 cells were gifts from Dr. Rosemarie Schmandt (The University of Texas MD Anderson Cancer Center, Houston, TX, USA); A2780-luc, ALST, HEYA8, OVCAR-3, OVCA420, OVCA432, TOV21G, RMG1, and OVCAR-5 cells were gifts from Dr. Samuel Mok (MD Anderson); and KOC7C and SMOV2 cells were gifts from Dr. Naoto Ueno (MD Anderson). ES-2, SKOV3 and TOV21G were originally from ATCC (Manassas, VA, USA). All cell lines were authenticated by the MD Anderson Characterized Cell Line Core by STR (Short Tandem Repeat Polymorphism) profiling.

### 4.2. Chemotherapeutic Agents

Cisplatin (Pfizer, New York, NY, USA), carboplatin (Hospira, Lake Forest, IL, USA), and paclitaxel (Hospira, Lake Forest, IL, USA) were obtained in suspension from the MD Anderson pharmacy.

### 4.3. Gene Expression Profiling

A2780 cells were treated with 5 or 10 μM cisplatin (Teva, Irvine, CA, USA), and total RNA was extracted from the cells at 0, 0.5, 1, 2, and 16 h after treatment using a mirVana miRNA isolation kit (Ambion, Austin, TX, USA). Similarly, OVCA420 cells were treated with 2.5 and 5 μM cisplatin for 16 h. To prepare samples of total RNA for hybridization to a GeneChip Human Genome U133 Plus 2.0 Array (Affymetrix, Santa Clara, CA, USA), a MessageAmp Premier RNA amplification kit (Thermo Fisher Scientific, Waltham, MA, USA) was used following the manufacturer’s protocol. Briefly, 100 ng of total RNA was reverse-transcribed to synthesize first-strand cDNA. After second-strand cDNA synthesis, an in vitro transcription reaction generated biotin-labeled antisense RNA. Next, 12 μg of a biotin-labeled antisense RNA sample was fragmented and hybridized to a U133 Plus 2.0 array in a GeneChip 640 hybridization oven at 45 °C for 16 h. The arrays were washed and stained using a Fluidics Station 450 and scanned using a confocal laser GeneChip Scanner 3000. CEL data files generated by the GeneChip Human Genome U133 Plus 2.0 Array were imported into the dChip software program for normalization of expression signals using an invariant set algorithm for identification of differentially expressed genes as described previously [43].

### 4.4. Semiquantitative Reverse Transcriptase-Polymerase Chain Reaction (RT-PCR)

To measure expression of genes of interest, total RNA was extracted from cell lines using a PureLink RNA mini kit (Invitrogen, Carlsbad, CA, USA) following the manufacturer’s protocol, including treatment of extracted total RNA with DNase treatment. For RNA extraction from mouse tissues, frozen tissue samples were cut and stored for at least 16 h in RNAlater-Ice (Thermo Fisher Scientific, Waltham, MA, USA) at −20 °C. After tissue transition, total RNA was isolated from the tissue using the mirVana miRNA isolation kit following the manufacturer’s protocol. The DNA-free kit was then used to remove the genomic DNA from the extracted total RNA. Once RNA was extracted using either of these kits, 1 μg RNA was reversed transcribed using the High-Capacity cDNA Reverse Transcription Kit (Applied Biosystems, Carlsbad, CA, USA) according to the manufacturer’s protocol. RT-PCR was performed as follows: 10 μL of Master Mix (7.5 μL of 2× iQ Supermix; Bio-Rad, Hercules, CA, USA), 1.75 μL of molecular grade water, and 0.75 μL of 20× TaqMan gene expression assay mix (Applied Biosystems, Foster City, CA, USA) were added to 5 μL of diluted (1:25) cDNA. RT-PCR was performed using a C1000 thermal cycler (Bio-Rad, Hercules, CA, USA). All TaqMan assays used were bought from Applied Biosystems. Cyclophilin A (PPIA) and beta-glucuronidase (GUSB) were used as controls.

### 4.5. Western Blot Assay

Total protein was extracted either from cell lines or tumor tissue using RIPA buffer (Sigma-Aldrich, St. Louis, MO, USA) containing protease inhibitors. Thirty micrograms of protein was subjected to electrophoresis on a 10% sodium dodecyl sulfate-polyacrylamide gel and transferred to a nitrocellulose membrane. After being blocked for 1 h in 5% milk in phosphate-buffered saline (PBS) with 0.05% Tween 20, the membrane was incubated overnight at 4 °C with a primary antibody, rinsed three times with PBS with 0.05% Tween 20 for 10 min each, and incubated at room temperature for 1 h with the secondary antibody. After being further rinsed three times for 10 min each, the membrane was scanned using an Odyssey imaging machine (LI-COR Biosciences, Lincoln, NE, USA). The primary antibodies used were anti-nonsteroidal anti-inflammatory drug-activated gene/placental transformation growth factor-β, rabbit polyclonal (anti-GDF15, 1:1000; Millipore, Billerica, MA, USA), and anti-β-actin (1:3000; Sigma-Aldrich, St. Louis, MO, USA) antibodies. The secondary antibodies used were goat anti-mouse and goat anti-rabbit antibodies (1:10,000; LI-COR Biosciences, Lincoln, NE, USA).

### 4.6. Stable GDF15 Short Hairpin RNA Knockdown

Two different GDF15 mission shRNA lentiviral transduction particles (catalog # TRNCN0000058388, TRNCN0000058389, Sigma-Aldrich, St. Louis, MO, USA) were transfected into the A2780 in a 6-well plate 24 h after plating to knockdown the expression of GDF15 in A2780 cells as two independent stable GDF15-KD A2780 cell lines. Mission pLKO.1-puro non-mammalian shRNA control transduction particles (catalog #SHC002V, Sigma-Aldrich, St. Louis, MO, USA) was transfected into A2780 cells as GDF15-NT A2780 control cell line. Puromycin (InvivoGen, San Diego, CA, USA) was used for stable selection at a concentration of 1 µg/mL. These stable cell lines were used in the mice studies.

### 4.7. GDF15 Secretion in a Blood Mouse Experiment

All animal studies were approved by the MD Anderson Institutional Animal Care and Use Committee and carried out under protocol #00000377-RN00 and 00000913-RN00. All nude mice were obtained from MD Anderson Experimental Radiation Oncology Breeding Core with approved animal protocols. No human subjects were involved in this study. The animal protocol# 00000377-RN00 and 00000913-RN00 was approved on 4 January 2011 and 3 January 2014 respectively by the IACUC committee. Mice were housed in a modified barrier room at the Research Animal Support Facility (RASFH) at MD Anderson. The Department of Veterinary Medicine and Surgery (DVMS) maintains the RASFH with AAALAC accreditation standards. Fifty 5- to 6-week-old female nude mice were injected intraperitoneally with 2 × 10^6^ A2780-luc cells (cisplatin-sensitive) or 5 × 10^6^ RMG1-luc cells (cisplatin-resistant). The mice were then monitored weekly for tumor growth via injection of D-luciferin (Caliper Life Sciences, Hopkinton, MA, USA) and use of a Xenogen imaging system (Caliper Life Sciences). Once tumors developed in the mice, 25 mice were administered PBS (*n* = 5), 2.5 mg/kg cisplatin (*n* = 10), or 5 mg/kg cisplatin (*n* = 10) via intraperitoneal injection. The cisplatin doses chosen are based on previous systematic determination of the maximum tolerated dose (~6 mg/kg) of cisplatin treatment in mice such that doses less than the maximum tolerated dose will have minimal or no impact on body weight and the development of clinical signs [44]. Mice with tumor burden had no sign of weight loss, loss of locomotion or any sign of moribund) at the time of treatment. Twenty-four or 48 h after treatment, five mice were randomly selected to be euthanized at each time point for both cisplatin concentrations according to Institutional Animal Care and Use Committee guidelines. CO_2_ chambers were operated according to specific instructions provided by the Departments of Veterinary Medicine and Surgery (specified CO_2_ injection setting; chambers will not be overcrowded and animals from different cages will not be mixed together). CO_2_ was used as the primary method of euthanasia followed by cervical dislocation as secondary means of ensuring death. Tumor and blood samples were collected from the mice. A week after the first 25 mice were given treatment, the experiment was repeated with the remaining 25 mice.

### 4.8. GDF15 Knockdown in Mice

All nude mice were obtained from MD Anderson Experimental Radiation Oncology Breeding Core after MD Anderson Institutional Animal Care and Use Committee approval of the study. Six-week-old female nude mice were injected intraperitoneally with 2 × 10^6^ A2780 control or GDF15-knockdown (KD) A2780 cells following Institutional Animal Care and Use Committee approved protocol. On days 18 and 23 after injection of the cells and confirmation of tumor formation, the mice (10 per treatment group) were given 5 mg/kg cisplatin or PBS via intraperitoneal injection. Forty-eight hours after the last cisplatin injection, mice were euthanized according to Institutional Animal Care and Use Committee guidelines. CO_2_ was used as the primary method of euthanasia followed by cervical dislocation as secondary means of ensuring death. Tumor and blood samples were then collected from each mouse. RNA and protein were extracted from the mouse tumor samples. Sera from the blood was used for the enzyme-linked immunosorbent assay (ELISA).

### 4.9. RNAseq Analysis

Tumor tissues from orthotopic mouse model were stored in RNAlater solution until extraction with Qiagen RNeasy Mini Kit. Sequencing libraries were prepared using KAPA Stranded RNA-Seq Kit (Roche Diagnositcs, Indianapolis, IN, USA) and run in a single lane using Illumina HiSeq4000 instrument. A total of eight tumor samples (Two GDF15-KD A2780 tumors and two GDF15-NT A2780 tumors from mice treated with PBS or cisplatin) were analyzed by RNAseq. Sequencing reads (Fastq files) from the RNAseq were analyzed using CLC Genomics Workbench version 11 to identify differentially expressed genes. Reads were mapped to human reference genome GRCH38 as well as to mouse reference genome GRCM38 with default setting and gene expression was estimated with the EM estimation algorithm and reported as TPM (transcripts per million). The RNAseq data has been deposited into GeoArchive (GEO accession #GSE132610).

### 4.10. ELISA

When the mice were killed, blood was collected from them and stored in serum tubes. The blood was allowed to clot for at least 30 min at room temperature and then centrifuged at full speed for 10–15 min at 4 °C. After centrifugation, the serum was transferred to a centrifuge tube and stored at −80 °C until all blood samples were collected. A human GDF15 Quantikine enzyme-linked immunosorbent assay kit (R&D Systems, Minneapolis, MN, USA) was used to measure GDF15 levels in the sera of mice according to the manufacturers’ protocol. Absorbance was measured using a BMG LABTECH plate reader. Absorbance at 540 nm along with 450 nm was used for correction. Prism version 6 (GraphPad Software, La Jolla, CA, USA) was used to analyze the data.

### 4.11. Immunohistochemistry

Immunohistochemical analysis of paraffin-embedded tissue samples from the GDF15-KD mice was conducted. Briefly, slides were deparaffinized and dehydrated. Antigen retrieval was conducted in a decloaking chamber (Biocare Medical, Concord, CA, USA) at 121 °C for 3 min and 95 °C for 1 minin citrate buffer (Poly Scientific, Bay Shore, NY, USA). Slides were stained with an anti-Ki67 mouse monoclonal antibody (catalog #550609; BD Pharmingen, San Jose, CA, USA) at a dilution of 1:50 and counterstained with hematoxylin using a Lab Vision Autostainer 360 (Thermo Fisher Scientific, Waltham, MA, USA). The mouse-on-mouse HRP-polymer (Biocare Medical, Pacheco, CA, USA) was also used in this procedure.

Slides stained for Ki67 were then analyzed with the assistance of the MD Anderson Flow Cytometry and Cellular Imaging facility and a Vectra Quantitative Pathology System. Images of the stained slides were taken using a Vectra 2 microscope along with the in Form 2.2 software program (PerkinElmer, Waltham, MA, USA) for quantification of the percentage of Ki67-positive tumor cells and examination of the tumor composition. A few immune cells were also Ki67-positive. Thirty randomly chosen fields at 20× magnification were captured for image analysis. The software was set up to detect tissue segmentation (e.g., tumor cells, vascular and fibrous region, other cell types, noncellular region based on cellular, cytoplasmic, Ki-67 staining and nuclear features) (Figure S1), cell segmentation, and staining intensity. Images that did not contain any tissue or tissue that did not include any tumor cells were rejected and not included in the analysis. The percentage of tumor cells with Ki67-positive nuclear staining was calculated by counting an average of 26,900 total tumor cells in each tumor sample. The ovarian tumor composition (tumor-stroma ratio) was calculated using tissue segmentation data.

### 4.12. RPPA

Protein was extracted from 20–40 μg of mouse tumor samples in RIPA buffer obtained from the Functional Proteomics Reverse Phase Protein Array (RPPA) Core facility at MD Anderson. Frozen tumor samples were homogenized in 200 μL of ice-cold RIPA buffer. Samples were then spun down for 5 min at 2600 rpm and frozen for at least 1 h before continuing with protein isolation. Samples were thawed and spun down at full speed for 20 min at 4 °C. The protein concentration was determined using a Bio-Rad Protein Assay. Samples were then mixed in 4× sodium dodecyl sulfate buffer without bromophenol blue and boiled for 5 min. Next, samples were submitted to the Functional Proteomics Reverse Phase Protein Array (RPPA) Core facility for further processing at a concentration of 1 μg/μL. Serially diluted protein lysates were arrayed on nitrocellulose-coated slides (Grace Bio-Labs, Bend, OR, USA) using an Aushon 2470 Arrayer (Aushon BioSystems, Billerica, MA, USA). Each slide was probed using a previously validated primary antibody and biotin-conjugated secondary antibody. Sixty mouse anti-human IgG primary antibodies and 214 rat or goat anti-human IgG primary antibodies were used. Amplification of the original obtained signal was performed using a Dako Cytomation-catalyzed system (Dako, Santa Clara, CA, USA), and visualization of positivity was conducted using a DAB calorimetric reaction. To generate spot intensity, the slides were scanned, analyzed, and quantified using the customized software program MicroVigene (VigeneTech Inc., Carlisle, MA, USA). The SuperCurve fitting logistic model developed by the MD Anderson Department of Bioinformatics and Computational Biology (https://bioinformatics.mdanderson.org/public-software/supercurve/) was used to fit each dilution curve. Data points were normalized for protein loading and transformed to normalized linear values at the protein expression level.

### 4.13. Statistical Analysis

The Kruskal-Wallis test was used for analysis of GDF15 secretion in mouse blood experiments for both mice sera and the RT-PCR analysis from tumor samples. The Mann-Whitney U test was used for all analyses conducted as a result of the GDF15-KD experiment. Unless otherwise stated, the error bars represent standard error. Prism version 6 (GraphPad Software, La Jolla, CA, USA) was used to conduct the analyses.

## 5. Conclusions

Up-regulation of GDF15 in ovarian cancer cells with wild-type p53 can modulate the canonical pathways in the tumor microenvironment in response to cisplatin.

## Supplementary Materials

The following are available online at https://www.mdpi.com/2072-6694/12/10/3043/s1, Figure S1: Images of control GDF15-NT A2780 and GDF15-KD A2780 ovarian tumor sections subjected to tissue segmentation analysis using the Vectra 2 quantitative pathology imaging software program. Tumor cell regions are highlighted in red, and stromal regions are highlighted in yellow (vascular and fibrous regions), green (other cell types) or blue (noncellular region), Figure S2: Validation of GDF15 knock down in vivo and in vitro: (a) Semiquantitative RT-PCR analysis of GDF15 expression performed using total RNA extracted from A2780 cells with control nontarget short hairpin RNA and GDF15-KD A2780 cells with two GDF15 short hairpin RNAs (KD1 and KD2); (b) Western blot analysis of induction of GDF15 protein expression by treatment with cisplatin in GDF15-KD A2780 cells; (c) Semiquantitative RT-PCR analysis of GDF15 expression performed using total RNA extracted from mouse tumors formed with control A2780-NT cells and GDF15-KD cells; (d) Enzyme-linked immunosorbent assay of GDF15 sera levels in mice with tumors formed from control A2780-NT cells and GDF15-KD cells. *** *p* < 0.0002, Figure S3: Mouse GDF15-KD A2780 tumors had markedly lower endogenous IgG mouse antigens than that in A2780 tumors based on RPPA analysis with 274 antibodies. The rat or Goat anti-human antibodies (*n* = 214) detected only human specific proteins, while the mouse anti-human antibodies (*n* = 60) detected both mouse IgG and the human specific proteins, Figure S4: Original images of western blots, Table S1: Differentially expressed genes in cisplatin treated A2780 cells with wild-type p53, Table S2: The number of sequencing reads mapped to mouse genome GRCm38 from RNAseq analysis of xenograft tumors, Table S3: Differentially expressed genes from GDF15-NT A2780 tumors from mice treated with cisplatin versus PBS using RNAseq data mapping to human Genome GRCH38, Table S4: Differentially expressed genes from GDF15-KD A2780 tumors from mice treated with cisplatin versus PBS using RNAseq data mapping to human Genome GRCH38, Table S5: Differentially expressed genes from GDF15-NT A2780 tumors from mice treated with cisplatin versus PBS using RNAseq data mapping to mouse Genome GRCm38, Table S6: Differentially expressed genes from GDF15-KD A2780 tumors from mice treated with cisplatin versus PBS using RNAseq data mapping to mouse Genome GRCm38.
