# Supplementary material for: The Role of GDF15 in Regulating the Canonical Pathways of the Tumor Microenvironment in Wild-Type p53 Ovarian Tumor and Its Response to Chemotherapy
Source: Cancers (Basel). 2020 Oct 19;12(10):3043. doi: 10.3390/cancers12103043 (PMC7650722; doi:10.3390/cancers12103043)
Supplement: Supplementary file 1 [file cancers-12-03043-s001.zip › Supplemantary File/cancers-944995-supplementary.pdf]

Supplementary Materials:

The Role of GDF15 in Regulating the Canonical Pathways of the Tumor Microenvironment in Wild-Type p53 Ovarian Tumor and Its Response to Chemotherapy

Daisy I. Izaguirre, Chun-Wai Ng, Suet-Yan Kwan, Eucharist H. Kun, Yvonne T. M. Tsang, David M. Gershenson and Kwong-Kwok Wong

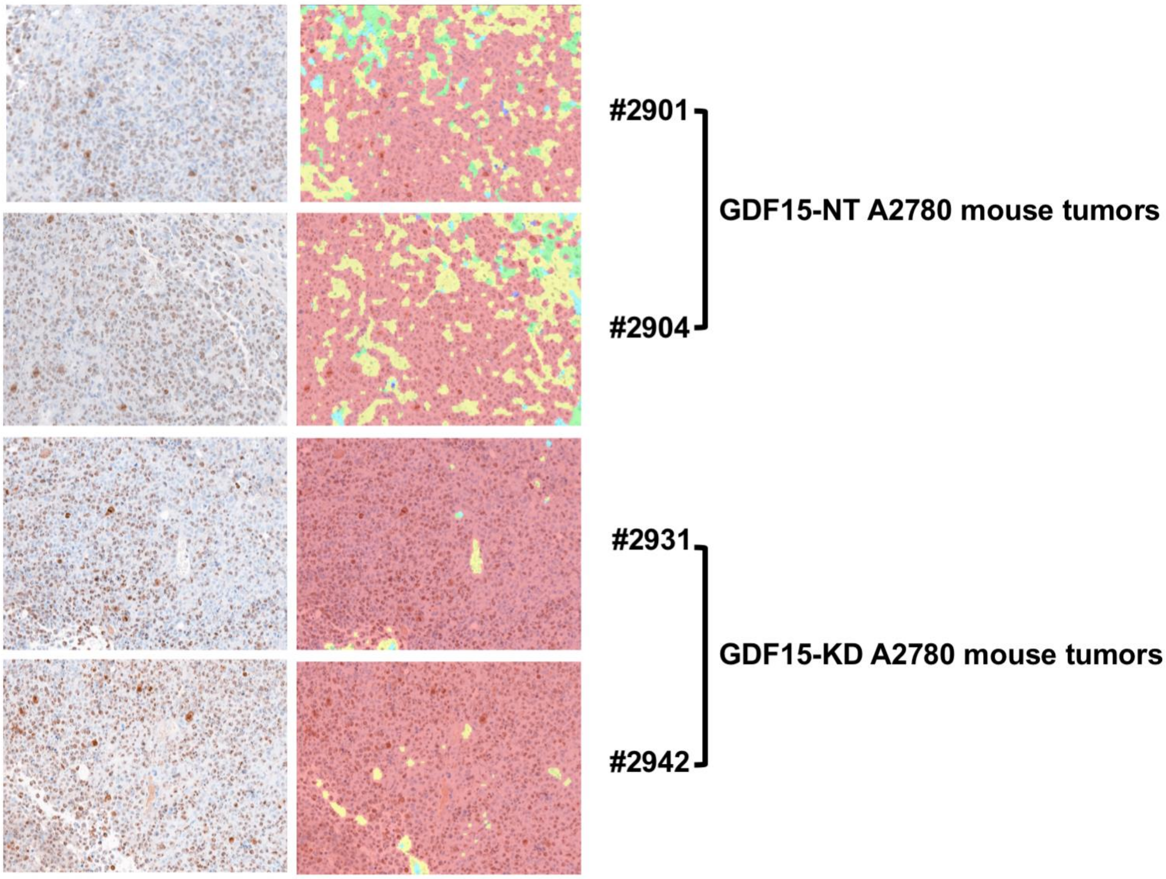

Figure S1. Images of control GDF15-NT A2780 and GDF15-KD A2780 ovarian tumor sections subjected to tissue segmentation analysis using the Vectra 2 quantitative pathology imaging software program. Tumor cell regions are highlighted in red, and stromal regions are highlighted in yellow (vascular and fibrous regions), green (other cell types) or blue (noncellular region).

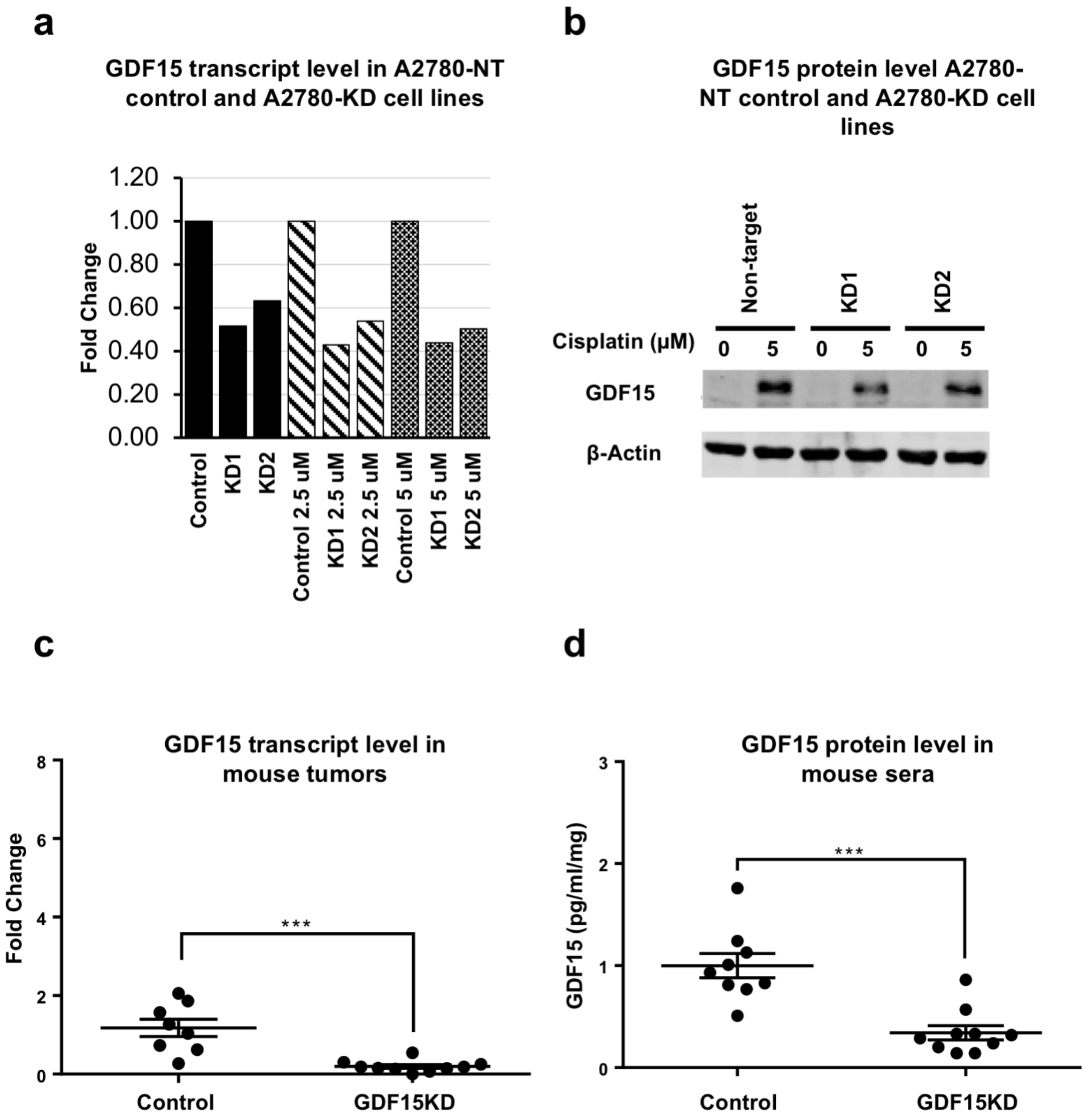

Figure S2. Validation of GDF15 knock down in vivo and in vitro: (a) Semiquantitative RT-PCR analysis of GDF15 expression performed using total RNA extracted from A2780 cells with control nontarget short hairpin RNA and GDF15-KD A2780 cells with two GDF15 short hairpin RNAs (KD1 and KD2); (b) Western blot analysis of induction of GDF15 protein expression by treatment with cisplatin in GDF15-KD A2780 cells; (c) Semiquantitative RT-PCR analysis of GDF15 expression performed using total RNA extracted from mouse tumors formed with control A2780-NT cells and GDF15-KD cells; (d) Enzyme-linked immunosorbent assay of GDF15 sera levels in mice with tumors formed from control A2780-NT cells and GDF15-KD cells. ***$p < 0.0002$.

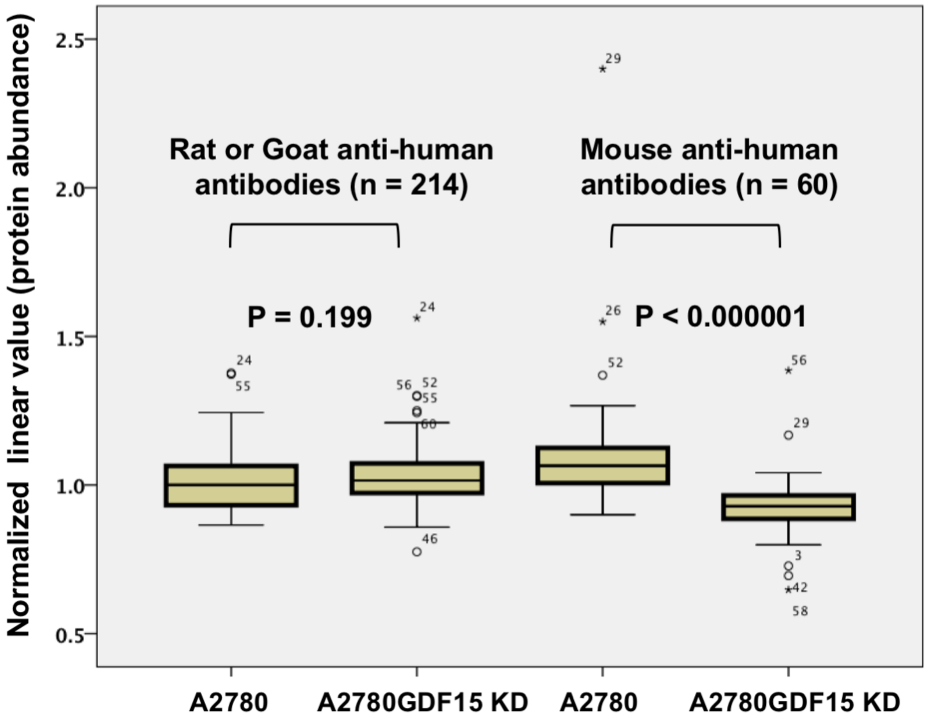

Figure S3. Mouse GDF15-KD A2780 tumors had markedly lower endogenous IgG mouse antigens than that in A2780 tumors based on RPPA analysis with 274 antibodies. The rat or Goat anti-human antibodies (n = 214) detected only human specific proteins, while the mouse anti-human antibodies (n = 60) detected both mouse IgG and the human specific proteins.

Figure 1 C

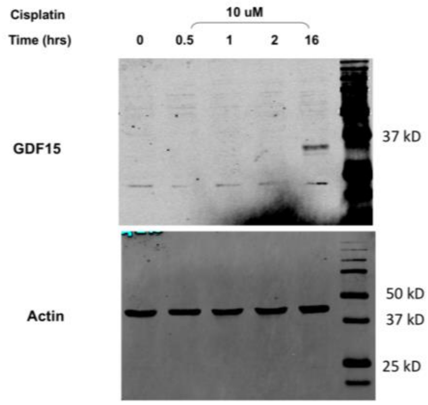

Figure 2b

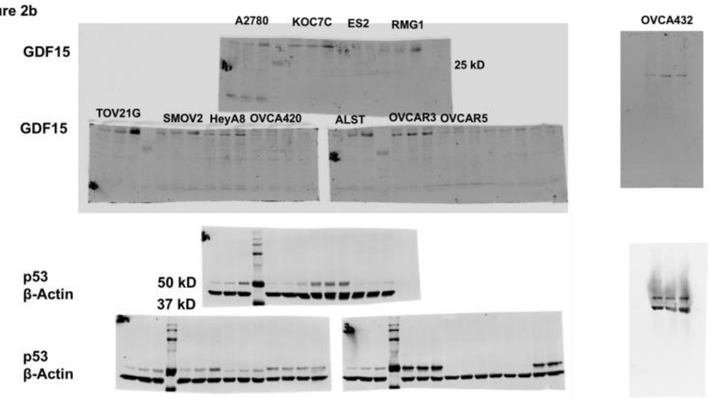

Using the 37 kD maker, the membranes were cut into upper half for p53 and β-Actin and lower half for GDF15 detection.

Figure 2d

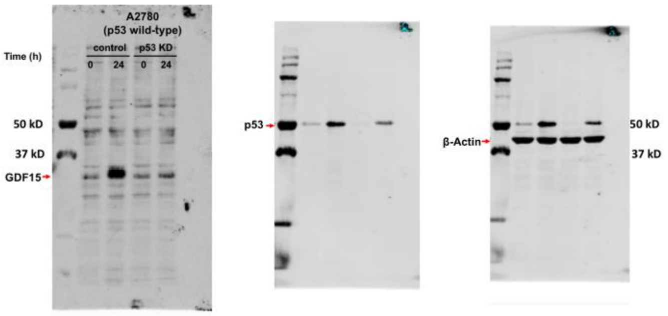

Figure 3

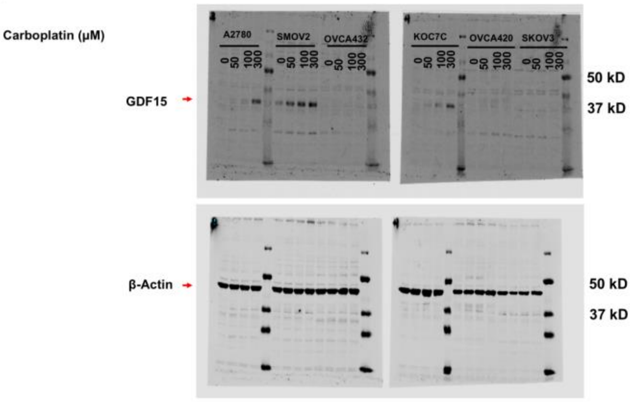

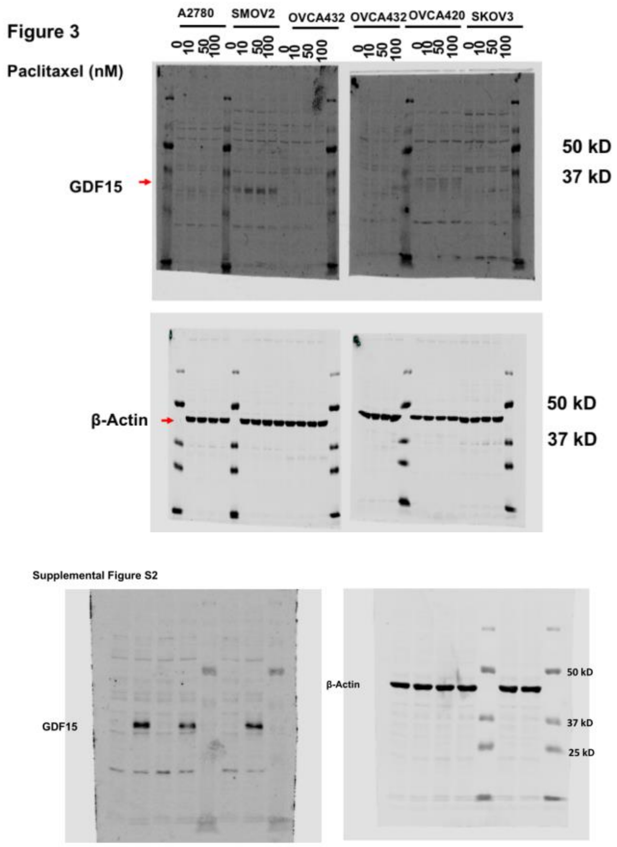

Figure S4. Original images of western blots.
